# Adult-Onset Still’s Disease Presenting as Antibiotic-Refractory Sepsis

**DOI:** 10.7759/cureus.93933

**Published:** 2025-10-06

**Authors:** Mohammed Alnims, Ahmer A Longi, Misbah Fazlani, Arsheena Mohamed, Ahmed Saleh

**Affiliations:** 1 Department of Internal Medicine, Mediclinic Welcare Hospital, Dubai, ARE; 2 Department of Internal Medicine, Mediclinic Welcare Hosptial, Dubai, ARE

**Keywords:** adult-onset still’s disease, adult-onset still’s disease (aosd), hyperferritinemia, il-6 blockade, tocilizumab, yamaguchi criteria

## Abstract

Adult-onset Still’s disease (AOSD) is a rare autoinflammatory disorder typified by quotidian fevers, an evanescent salmon-colored rash, arthralgia, neutrophilic leukocytosis, and markedly elevated inflammatory markers. Diagnosis is clinical and by exclusion, supported by classification frameworks like the Yamaguchi criteria. A previously healthy lady in her 20s presented with fever above 39°C, severe generalized myalgia, pharyngitis, a transient salmon-colored rash, neutrophilic leukocytosis, and markedly elevated inflammatory markers. Competing infectious, autoimmune, and malignant etiologies were excluded. Based on clinical features and Yamaguchi criteria, AOSD was suspected. She improved rapidly with intravenous methylprednisolone pulse therapy. During steroid taper as an outpatient, she developed a biochemical flare with ferritin rising to 8,807 ng/ml; tocilizumab was initiated for disease control. This case underscores prompt recognition of AOSD after exclusion of mimics, the interpretive value of ferritin trends, and successful escalation to interleukin-6 (IL-6) blockade for relapse, consistent with emerging data emphasizing targeted cytokine inhibition.

## Introduction

Adult-onset Still’s disease (AOSD) is a rare systemic inflammatory disorder characterized by dysregulated innate immunity and excessive cytokine signaling. Clinically, patients classically exhibit high spiking (often quotidian) fevers, a transient salmon-colored exanthem, sore throat, arthralgia/arthritis, and neutrophilic leukocytosis, usually accompanied by striking elevations in acute-phase reactants and ferritin [1]. As there is no single best test in diagnostics, AOSD remains a diagnosis of exclusion after thorough evaluation for infection, malignancy, and alternative rheumatic diseases.

Classification frameworks aid clinical diagnosis. The widely used Yamaguchi criteria prioritizes fever, typical rash, arthralgia, and leukocytosis with neutrophilia as major items, alongside minor features such as sore throat, lymphadenopathy/splenomegaly, liver enzyme abnormalities, and negative antinuclear antibody (ANA)/rheumatoid factor (RF) [1]. The Fautrel criteria incorporate laboratory surrogates of the cytokine storm, most notably glycosylated ferritin ≤20%, and can perform well in the appropriate clinical context [1, 2].

Pathologically, the syndrome reflects activation of innate immune pathways with elevated interleukin-1 beta (IL‑1β), IL‑6, TNF‑α, and IL‑18 driving fever, neutrophilia, hepatosplenic inflammation, and hyperferritinemia. A meta-analysis shows circulating IL-18 levels are significantly higher in AOSD than in controls and correlate with ferritin and C-reactive protein (CRP), reinforcing its candidacy as a disease activity biomarker [3]. Although ferritin is frequently very high, normal ferritin values do not exclude AOSD and should be interpreted in tandem with clinical features and other markers [4].

The clinical spectrum is heterogeneous. Beyond systemic flares, AOSD may mimic inflammatory myopathy with severe myalgia despite normal or low creatine kinase (CK), present with neurologic disease such as aseptic meningitis, or manifest cardiac involvement, including pericarditis and, rarely, acute valvular dysfunction [5-7]. Dermatologic phenotypes can vary and include dermatomyositis-like eruptions [8]. Onset or flares may occur in the peripartum period, highlighting obstetric considerations for diagnosis and treatment [9].

Management is staged rapid induction with systemic glucocorticoids to control systemic inflammation, followed by early steroid-sparing therapy for relapsing or refractory disease. Increasing real-world experience supports targeted cytokine inhibition, especially IL-1 and IL-6 blockade, often alongside methotrexate, to reduce corticosteroid exposure and maintain remission [1, 10]. Our case illustrates this paradigm, with brisk steroid responsiveness and subsequent control followed by a biochemical flare requiring tocilizumab therapy with successful control.

## Case presentation

**Figure 1:**
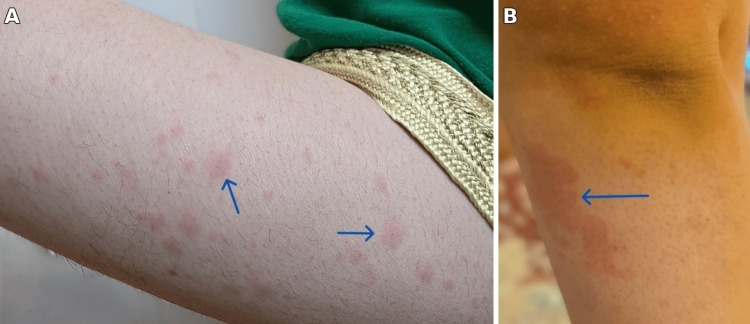
Salmon-colored rash on arms indicated by blue arrows in panes A and B

**Figure 2:**
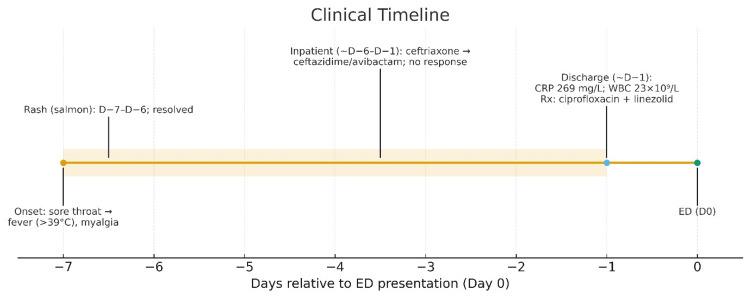
Timeline of symptoms and clinical course leading to her presentation

A lady in her 20s presented to the emergency department with a seven-day history of high-grade fever (> 39°C), severe generalized myalgia, throat discomfort, and a transient rash. The symptoms began with a sore throat, followed by her fever and body aches, predominantly affecting her upper limbs. She had developed a salmon-colored rash (Figure 1) during the first two days, which resolved before her presentation to our facility. Her past medical, family, and social histories were unremarkable. During a previous hospitalization at another facility, she was treated with intravenous ceftriaxone followed by broad-spectrum ceftazidime/avibactam without clinical improvement. At discharge from that facility, her CRP was 269 mg/L, and her white blood cell (WBC) count was 23 x 109/L. She was subsequently discharged on oral ciprofloxacin and linezolid (Figure 2).

On physical examination, the patient was alert and oriented but appeared weak. She was febrile (39°C). Mild proximal upper limb weakness was noted, with bilateral shoulder abduction graded at 4/5. She was hemodynamically stable and showed no signs of respiratory distress. No rash was present at the time of examination. Neurological and cardiopulmonary examinations were otherwise unremarkable. Routine laboratory tests were carried out (Table 1).

**Table 1:** The patient's laboratory investigations

Test	Result	Reference Range
C-reactive Protein (CRP)	342.0 mg/L	0.0 – 5.0 mg/L
White Blood Cells (WBC)	22.2 ×10⁹/L	4.0 – 11.0 ×10⁹/L
Neutrophils	91.4%	40 – 75%
Ferritin	1,528 ng/mL	13 – 120 ng/mL
Erythrocyte Sedimentation Rate (ESR)	71 mm/hr	2 – 39 mm/hr
Procalcitonin (PCT)	0.16 ng/mL	< 0.05 ng/mL
Hemoglobin	9.5 g/dL	11.5 – 16.0 g/dL
Platelets	530 ×10⁹/L	150 – 450 ×10⁹/L
Creatinine	25 µmol/L	53 – 97 µmol/L
Corrected Calcium	2.49 mmol/L	2.15 – 2.50 mmol/L
Creatine Kinase (CK)	14.8 U/L	34 – 145 U/L
Alanine Aminotransferase (ALT)	15.7 U/L	< 35 U/L
Aspartate Aminotransferase (AST)	22.9 U/L	< 35 U/L
Albumin	22.5 g/L	35 – 52 g/L
Alkaline phosphatase (ALP)	222 U/L	35 – 104 U/L
Total Bilirubin	13.8 µmol/L	< 21.0 µmol/L
Direct Bilirubin	9.81 µmol/L	< 7.0 µmol/L
Lactate Dehydrogenase (LDH)	243 U/L	135 – 214 U/L
Sodium (Na)	136.0 mmol/L	136 – 145 mmol/L
Potassium (K)	3.7 mmol/L	3.5 – 5.1 mmol/L
Bicarbonate (HCO₃)	20.0 mmol/L	22 – 28 mmol/L
Chloride (Cl)	96.2 mmol/L	98 – 107 mmol/L

Microbiology and immunology

Extensive microbiological testing was negative, including blood and urine cultures, dengue nonstructural protein 1 (NS-1) antigen and immunoglobulin M (IgM) antibodies, malaria smear, influenza rapid antigen test, *Brucella* IgM, and *Leptospira *IgM serologies. Autoimmune screening showed negative results for ANA, antineutrophil cytoplasmic antibodies (ANCA), and anti-cyclic citrullinated peptide (anti-CCP) antibodies, with a borderline positive PMSCL-100. Urine immunofixation for Bence-Jones proteins was also negative.

Imaging

**Figure 3:**
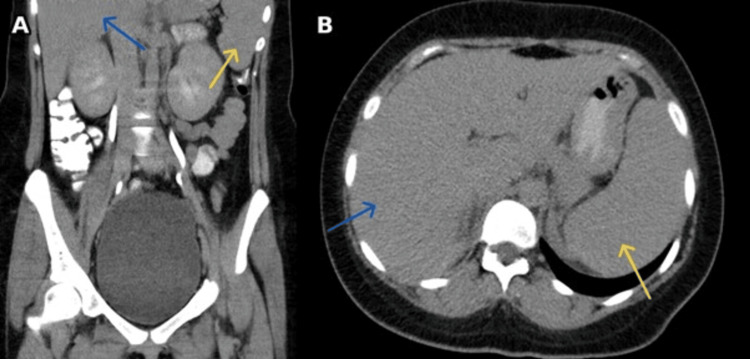
CT of the abdomen and pelvis with contrast Coronal section (A) and axial section (B) showing mild hepatosplenomegaly. The liver is indicated by blue arrows and the spleen is indicated by yellow arrows.

Contrast-enhanced computed tomography (CT) of the chest, abdomen, and pelvis (Figure 3) demonstrated mild hepatosplenomegaly, mesenteric lymphadenopathy, and bowel wall thickening consistent with colitis. Transthoracic echocardiography revealed normal cardiac function with mildly increased flow rates across the aortic valve but no evidence of vegetations or thrombus.

Diagnosis and management

Due to persistent inflammation and lack of response to broad-spectrum antibiotics, a rheumatology consultation was initiated. AOSD was suspected based on the Yamaguchi criteria. Concurrent evaluations by hematology were undertaken to exclude hematologic malignancies. A bone marrow trephine biopsy was non-diagnostic, while flow cytometry revealed no abnormal B-cell, T-cell, or myeloid populations. Cytology demonstrated trilineage hematopoiesis without evidence of malignancy. The patient was commenced on intravenous methylprednisolone pulse therapy, resulting in rapid and significant clinical improvement, including reduced myalgia, improved muscle strength, and a marked decrease in inflammatory markers. CRP levels dropped from 342 mg/L to 111 mg/L and subsequently to 60 mg/L. Procalcitonin declined to 0.06 ng/ml, and WBC count improved to 13 x 109/L. The patient remained hemodynamically stable and was discharged on a tapering regimen of oral corticosteroids.

Outcome and follow-up

Following discharge, during outpatient follow-up and while tapering corticosteroids, the patient experienced a recurrence of myalgia along with rising inflammatory markers with a marked increase in serum ferritin to 8,807 ng/mL. Considering this disease flare, tocilizumab was initiated. Hydroxychloroquine and methotrexate (10 mg once weekly) were also added for long-term disease control. Subsequently, the patient reported resolution of fever and musculoskeletal symptoms, with no recurrence of rash or joint pain. Her laboratory investigation also showed improvement in inflammatory markers (Table 2). She remained clinically stable on the adjusted regimen, with ongoing follow-up planned to monitor the disease activity and tailor immunosuppressive therapy accordingly.

**Table 2:** Serial laboratory investigations

Test	Labs on Follow-Up (Flare)	Post One Week of Tocilizumab	Post 2 Weeks of Tocilizumab	Reference Range
Erythrocyte Sedimentation Rate (ESR)	20 mm/hr	-	7 mm/hr	2-39 mm/hr
Alanine Aminotransferase (ALT)	-	21.8 U/L	15.4 U/L	<35 U/L
Aspartate Aminotransferase (AST)	-	21.7 U/L	19.3 U/L	<35 U/L
Creatinine	32.3 umol/L	31.6 umol/L	43.7 umol/L	53-97 umol/L
C-reactive Protein (CRP)	45.5 mg/L	5.5 mg/L	< 0.6 mg/L	0.0–5.0 mg/L
Ferritin	8807 ng/mL	429 ng/mL	98.7 ng/mL	30–120 ng/mL
Lactate Dehydrogenase (LDH)	857 U/L	267 U/L	231 U/L	135-214 U/L
Hemoglobin	10.7 g/dL	11.8 g/dL	12.4 g/dL	11.5-16.0 g/dL
White Cell Count	8.23 10^3^/uL	9.8 10^3^/uL	9.9 10^3^/uL	4-11 10^3^/uL
Platelets	106 10^3^/uL	678 10^3^/uL	433 10^3^/uL	150-450 10^3^/uL

## Discussion

The constellation of quotidian high-grade fever (> 39°C), transient salmon-colored rash, neutrophilic leukocytosis, pharyngitis, and abnormal liver enzyme tests (elevated alkaline phosphatase with hypoalbuminemia) fulfills the Yamaguchi (Table 3) framework (≥ 5 criteria with ≥ 2 major) after rigorous exclusion of infection, malignancy, and alternative rheumatic diseases [1]. Fautrel's criteria further emphasize glycosylated ferritin ≤20%, which was not measured here [1, 2]. Marked ferritin elevation at presentation with further surge during relapse supports disease activity. Notably, AOSD may present with normal ferritin; thus, dynamic trends and the full clinical picture are essential [4]. Given prominent myalgia and proximal weakness, inflammatory myopathy was considered; however, CK was low/normal, and the combination with reports that AOSD can mimic myositis while maintaining a normal CK [5]. 

**Table 3:** Yamaguchi's criteria Must meet ≥ 5 criteria, of which ≥ 2 must be major. Reference [1] WBC: white cell counts; PMN: polymorphonuclear cells; LFT: liver function test; RF: rheumatoid factor; ANA: antinuclear antibody

Major Criteria	Minor Criteria	Exclusion Criteria
Fever > 39^o^C > 1 Week	Sore Throat	Sepsis
Arthralgia/Arthritis > Weeks	Lymphadenopathy	Malignancy (Lymphoma)
Typical Rash	Hepatosplenomegaly	Vasculitis
WBC > 10000, 80% PMNs	Deranged LFTs	
	RF and ANA Negative	

Clinical spectrum and phenotype

AOSD encompasses systemic, intermittent/polycyclic, and chronic articular phenotypes. Systemic disease features quotidian fever, rash, and serositis; the chronic articular form predisposes to erosive arthritis. Our patient’s prominent systemic features and rapid steroid responsiveness fit a systemic phenotype with early relapse during taper [1, 10].

Diagnostic uncertainty frequently stems from overlap of infectious syndromes (bacterial sepsis, zoonoses such as brucellosis, leptospirosis, malaria, and dengue), hematologic malignancies (notably lymphoma), and connective tissue diseases. In our case, extensive microbiology was negative, imaging lacked focal infection, and bone marrow studies showed trilineage hematopoiesis without malignant populations. The presence of sore throat, transient rash, neutrophilia, and liver test abnormalities collectively supports AOSD when these mimics are excluded [1].

Literature highlights AOSD mimicking inflammatory myopathy with disproportionate myalgia yet normal CK levels; neurologic involvement such as aseptic meningitis; and rare cardiac lesions, including acute severe mitral regurgitation from flail leaflet [5-7]. Awareness of these variants prevents diagnostic delay. Variable cutaneous phenotypes, such as dermatomyositis-like eruptions, further expand the spectrum [8]. Although hyperferritinemia is common and often dramatic, its absence does not preclude AOSD. IL-18 correlates positively with CRP and ferritin and may assist in assessing activity or distinguishing flares from infection in selected settings [3]. Glycosylated ferritin ≤20% supports the Fautrel framework; however, limited availability constrains routine use [1, 2].

Macrophage activation syndrome (MAS) represents a fulminant hyperinflammatory complication at the AOSD-HLH interface and warrants early consideration when cytopenia, coagulopathy, hepatopathy, and extreme ferritin elevations accompany persistent fevers. While our patient did not meet MAS criteria, the significant ferritin surge during relapse mandated close surveillance. First-line therapy typically includes high-dose corticosteroids, with rapid escalation to cytokine blockade in refractory cases [1].

Contemporary practice favors a step-up approach beginning with glucocorticoids for induction, then introduction of steroid-sparing therapy in patients with relapse, dependence, or contraindications. Real-world cohorts document frequent and effective use of IL-1 inhibitors and methotrexate; accumulating experience supports IL-6 blockade (tocilizumab) in refractory systemic disease, with improvements in fever, CRP, and ferritin and facilitation of steroid taper [1, 10]. In this case, tocilizumab achieved clinical remission after a flare.

Prior to biologic therapy, screen for latent tuberculosis and hepatitis C. During IL-6 inhibition, monitor for infection, cytopenia, transaminase elevations, and dyslipidemia. Disease activity should be tracked through symptoms and inflammatory markers (CRP/ESR), supplemented by ferritin, and, where accessible, IL-18 to guide tapering and maintenance strategies [3, 10]. Cases of de novo AOSD or flares in the early postpartum period are reported. Diagnostic vigilance is required to differentiate from obstetric sepsis and other puerperal conditions. Medication choices should be individualized with obstetric input, considering fetal/neonatal safety during pregnancy and lactation [9].

Many patients achieve remission with appropriate therapy, yet a subset transitions to polycyclic or chronic-articular disease, emphasizing the importance of early steroid-sparing strategies and treat-to-target follow-up. Our patient’s favorable trajectory after IL-6 blockade is aligned with literature advocating targeted cytokine inhibition to maintain remission and minimize corticosteroid exposure [1, 10].

In cases of fever with unknown origin associated with pharyngitis, evanescent rash, neutrophilia, and elevated inflammatory markers, AOSD should be considered [1], even if CK levels are normal in the setting of severe myalgia. The ferritin should be interpreted in context, and the trend should be followed [3]. In case of worsening with infectious, malignancy, and other rheumatological causes excluded, early escalation to cytokine inhibitors for relapse or steroid dependence should also be considered [10]. Close vigilance needs to be done for MAS and organ-specific complications.

## Conclusions

AOSF should be suspected in young adults with spiking fevers, pharyngitis, transient salmon-colored rash, and neutrophilia after exclusion of infectious, malignant, and autoimmune mimics. Ferritin trends help gauge activity but are not definitive alone. Early glucocorticoid therapy is frequently effective; timely escalation to targeted cytokine inhibitors such as tocilizumab can secure sustained control in relapsing disease.
